# Mesenteric lymph nodes: a critical site for the up-regulatory effect of hUC-MSCs on Treg cells by producing TGF-β1 in colitis treatment

**DOI:** 10.1186/s13287-024-03809-x

**Published:** 2024-07-02

**Authors:** Qixiang Zhang, Zhu Zeng, Ning Wei, Yueyan Su, Jing Wang, Qi Ni, Yukai Wang, Jingwen Yang, Xiaoyan Liu, Huanke Xu, Guangji Wang, Yunlong Shan, Fang Zhou

**Affiliations:** 1 Key Laboratory of Drug Metabolism and Pharmacokinetics, Haihe Laboratory of Cell Ecosystem, State Key Laboratory of Natural Medicines, China Pharmaceutical University, Nanjing, China; 2 Jiangsu Renocell Biotech Co., Ltd, Nanjing, China; 3 Present Address: No. 639 Longmian Avenue, Nanjing, Jiangsu China; 4 Present Address: Tongjiaxiang #24, Nanjing, Jiangsu China

**Keywords:** Mesenchymal stem cells, Experimental colitis, Intraperitoneal injection, Mesenteric lymph nodes, TGF-β1, Treg cells

## Abstract

**Background:**

Mesenchymal stem cells (MSCs) demonstrate a wide range of therapeutic capabilities in the treatment of inflammatory bowel disease (IBD). The intraperitoneal injection of MSCs has exhibited superior therapeutic efficacy on IBD than intravenous injection. Nevertheless, the precise in vivo distribution of MSCs and their biological consequences following intraperitoneal injection remain inadequately understood. Additional studies are required to explore the correlation between MSCs distribution and their biological effects.

**Methods:**

First, the distribution of human umbilical cord MSCs (hUC-MSCs) and the numbers of Treg and Th17 cells in mesenteric lymph nodes (MLNs) were analyzed after intraperitoneal injection of hUC-MSCs. Subsequently, the investigation focused on the levels of transforming growth factor beta1 (TGF-β1), a key cytokine to the biology of both Treg and Th17 cells, in tissues of mice with colitis, particularly in MLNs. The study also delved into the impact of hUC-MSCs therapy on Treg cell counts in MLNs, as well as the consequence of *TGFB1* knockdown hUC-MSCs on the differentiation of Treg cells and the treatment of IBD.

**Results:**

The therapeutic effectiveness of intraperitoneally administered hUC-MSCs in the treatment of colitis was found to be significant, which was closely related to their quick migration to MLNs and secretion of TGF-β1. The abundance of hUC-MSCs in MLNs of colitis mice is much higher than that in other organs even the inflamed sites of colon. Intraperitoneal injection of hUC-MSCs led to a significant increase in the number of Treg cells and a decrease in Th17 cells especially in MLNs. Furthermore, the concentration of TGF-β1, the key cytokine for Treg differentiation, were also found to be significantly elevated in MLNs after hUC-MSCs treatment. Knockdown of *TGFB1* in hUC-MSCs resulted in a noticeable reduction of Treg cells in MLNs and the eventually failure of hUC-MSCs therapy in colitis.

**Conclusions:**

MLNs may be a critical site for the regulatory effect of hUC-MSCs on Treg/Th17 cells and the therapeutic effect on colitis. TGF-β1 derived from hUC-MSCs promotes local Treg differentiation in MLNs. This study will provide new ideas for the development of MSC-based therapeutic strategies in IBD patients.

**Supplementary Information:**

The online version contains supplementary material available at 10.1186/s13287-024-03809-x.

## Introduction

Inflammatory bowel disease (IBD), mainly including ulcerative colitis (UC) and Crohn’s disease (CD), has emerged as a global disease with no available cure [1, 2], which is characterized by recurrent chronic inflammation of the gastrointestinal tract [3, 4]. In the past decade, an expansive armamentarium of biologic and small-molecule therapies has become available for the induction and maintenance of remission in IBD [5]. Despite these advancements, approximately 30% of patients do not respond to biologicals and of those who do initially respond, 50% experience a relapse within one year. Additionally, the majority of medications used to treat IBD are immunosuppressive, which can result in a heightened susceptibility to infections and cancer [6]. Given the variability in individual responses to these drugs, a more diverse array of therapeutic approaches is necessary.

Mesenchymal stem cells (MSCs) have emerged as a valuable therapeutic approach in tissue repair and immunomodulation, offering numerous advantages. MSCs possess the capability to be attracted to areas of tissue damage or inflammation and modulate the immune microenvironment at the site of injury. The safety and efficacy of MSC-based therapy have been demonstrated in IBD patients, including those who are unresponsive to traditional clinical or biological treatments or are considered medically refractory [7–9]. Nevertheless, the intravenous injection of MSCs has been shown to result in inefficient homing to inflamed colon tissues, thereby limiting the optimal utilization of MSC therapy [10]. Although systemic infusion of MSCs remains a prevalent method for treating IBD [11], recent studies have demonstrated that intraperitoneal injections of MSCs exhibit greater efficacy in addressing colitis [10]. Nevertheless, the specific mechanisms underlying this phenomenon remain poorly understood. Further research is necessary to elucidate the relationship between the precise in vivo distribution of MSCs and their resulting biological effects following intraperitoneal injection.

An imbalance in the ratio of Treg to Th17 cells in the gut is a common characteristic typical of colitis. Therefore, upregulating Treg cells in the gut to restore balance between Treg and Th17 cells has been identified as a viable strategy for managing colitis [12–15]. It has been reported that MSCs induce Treg cell differentiation, which in turn limits the inflammatory response [16, 17]. Meanwhile, various investigations have indicated that human mesenchymal stem cells (hMSCs) possess the ability to release anti-inflammatory agents and have been shown to be dependent on local microenvironmental [18, 19]. TGF-β1 is of particular interest because it regulates T-cell-mediated tolerance and immunity through both Treg and Th17 cells in a context-dependent manner [20, 21]. Whether MSCs treatment could rebalance Treg/Th17 cells in colitis mice by inducing Treg cell differentiation need further investigation.

An important prerequisite for hMSCs to immune regulate by producing anti-inflammatory agents is migration to diseased tissues [22]. However, most studies have found MSCs administered systemically are predominantly distributed in the lungs, liver, and spleen after systemic infusion, with minimal distribution in the colon. In contrast, intraperitoneally injected MSCs could migrate into the inflamed colon and effectively reduced the amount of collagen deposition, the rate of epithelial cell apoptosis and the local production of pro-inflammatory cytokines [23, 24]. Here, we aim to explore the precise in vivo distribution of MSCs and their biological consequences focusing on the balance of Treg/Th17 cells especially in mesenteric lymph nodes (MLNs).

## Materials and methods

### Animal study

Male C57BL/6 mice (6–8 weeks) and male BALB/c mice (6–8 weeks) and were both purchased from the Beijing Vital River Laboratory Animal Technology Co (Beijing, China). All animals were maintained under the specific-pathogen free (SPF) condition with a constant temperature (23 ± 1.5˚C) and humidity (70 ± 20%) on a 12-hour light/dark cycle. All animal experiments were performed in compliance with Guidance for Care and Use of Laboratory Animals and approved by the Ethics Committee of the Experimental Animal Center at China Pharmaceutical University (SYXK2021-0011) and ARRIVE (Animal Research: Reporting of In Vivo Experiments) 2.0 guidelines. The animals were humanely euthanized using carbon dioxide inhalation according to institutional guidelines.

### hUC-MSCs preparation

The hUC-MSCs were manufactured by Jiangsu Renocell Biotech Co., Ltd. (Nanjing, China). The hUC-MSC product were identified by the minimal criteria suggested by International Society for Cellular Therapy (ISCT): (1) plastic adherent under tissue culture flask; (2) > 95% of the cell population expressed CD105, CD73, and CD90, and these cells were lack expression (< 2% positive) of CD45, CD34, CD11b, CD19, and HLA-DR as measured by flow cytometry (BD, FACS Calibur, USA); (3) differentiation potential into osteoblasts, adipocytes, and chondroblasts under standard in vitro differentiating conditions. The cell product has been certified by the National Institutes for Food and Drug Control of China.

### Induction of colitis by DSS

Colitis in male C57BL/6 mice was induced by providing drinking autoclaved water containing 3% (*w/v*) Dextran sulfate (DSS) (36000-50,000 kDa; MP Biomedicals, USA), *ad libitum* for 7 days, followed by regular drinking water [25].

### Treatment of colitis mice

At day 3 of DSS induction, mice were treated with hUC-MSCs (2 × 10^6^ cells/mice, 200 µL in volume). To prevent confounding bias, the placement of each mouse cage was randomized after cell transplantation. Then DSS induction was continued until day 7. At the therapeutic endpoint of hUC-MSCs, animals from each group were euthanized, colon lengths and spleen weights were measured, and samples were taken for macroscopic analysis.

To compare the efficacy of hUC-MSCs administered intravenously and intraperitoneally on DSS-induced colitis mice, 32 mice were divided into four groups (*n* = 8): control mice, DSS-induced colitis mice, DSS-induced colitis mice with intravenous injection of hUC-MSCs (i.v.) and DSS-induced colitis mice with intraperitoneal injection of hUC-MSCs (i.p.). To further investigate the effect of intraperitoneal administration of hUC-MSCs on intestinal inflammatory injury, 15 mice were divided into three groups (*n* = 5): control mice, DSS-induced colitis mice, and DSS-induced colitis mice with intraperitoneal injection of hUC-MSCs. To study the effect of hUC-MSCs with *TGFB1* Knockdown (hUC-MSCs^*TGFB1 KD*^) on DSS-induced colitis, 20 mice were divided into four groups (*n* = 5): control mice, DSS-induced colitis mice, DSS-induced colitis mice intraperitoneal injection of hUC-MSCs^*NC*^ (Negative Control) and DSS-induced colitis mice intraperitoneal injection of hUC-MSCs^*TGFB1 KD*^. To prevent confounding bias, the placement of each mouse cage was randomized.

### Distribution of hUC-MSCs

At day 7 of DSS induction, mice were randomized and injected intraperitoneally with hUC-MSCs (2 × 10^6^ cells/mice, 200 µL in volume). 30 mice were divided into two groups: control mice injected with hUC-MSCs and DSS-induced colitis mice injected with hUC-MSCs. Three time points in each group for sample collection after receiving hUC-MSCs injection were: 1 day, 3 days, and 7 days (*n* = 5 per group at every time point). At the indicated time points, animals were euthanized, and then different organs were collected for detecting the hUC-MSCs numbers. To prevent confounding bias, the placement of each mouse cage was randomized.

### Method for detecting hUC-MSCs in mouse tissues

We have established a sensitive, specific, and reliable qRT-PCR (Quantitative real time polymerase chain reaction) method based on the *Alu* gene [26–28]. The primers were designed targeting the human-specific unique sequence of *Alu*, the primer set for human-specific *Alu* repeat was as follows: forward, 5’- CACTACGCCCGGCTAATTT-3’; reverse, 5’-GCCTGTAATCCCAGCACTTT-3’. Total genomic DNA (gDNA) from mouse tissue was extracted with the FastPure Cell/Tissue DNA Isolation Mini Kit (Vazyme, Nanjing, China) based on the manufacturer’s protocol. The concentration and purity of total gDNA were detected by the Colibri Spectrophotometer (Berger, Germany). Then, the qRT-PCR assay of gDNA was performed with SYBR Green Mix (Bio-Rad, California, USA).

The standard curve of cycle threshold values (Ct) versus cell number (logarithmic form) was obtained through qRT-PCR amplification. Each test tissue was assayed alongside its respective standard curve. The concentration of hUC-MSCs in the tissue was determined by substituting the Ct value of the cDNA into the corresponding tissue’s standard curve. The precision and accuracy of this method have been verified for preclinical evaluation (Data not shown).

### Imaging of RFP-hUC-MSCs in mice vivo

DSS-induced colitis mice were injected intraperitoneally with RFP-hUC-MSCs (2 × 10^6^ cells/mice, 200 µL in volume). At the 1 day after injected with RFP-hUC-MSCs, animals of each group were euthanized and selected organs were collected. Organs were washed by PBS buffer (Phosphate Buffered Saline), and fluorescence intensities were measured by an IVIS with filter set as follows: Excitation/Emission 594/647 nm. Two different experimental groups were established: control mice injected with RFP-hUC-MSCs and DSS-induced colitis mice injected with RFP-hUC-MSCs. Each group comprised three mice, a total of six mice used during the experiment.

### **Enzyme-linked immunosorbent assay** (**ELISA**)

Fresh tissue homogenization and serum from mice were collected and stored at -80 °C until cytokine determination. Cytokine in tissue homogenization and serum were determined by ELISA Kits according to the manufacturer’s instructions. TGF-β1 ELISA Kits were purchased from Elabscience Biotechnology Co., Ltd (Wuhan, China). TNF-α, IL-1β, IFN-γ and IL-6 ELISA Kits were purchased from CUSABIO Co., Ltd (Wuhan, China).

For the determination of TGF-β1 concentration in tissues, 60 mice were divided into four groups: control mice, control mice injected with hUC-MSCs, DSS-induced colitis mice and DSS-induced colitis mice injected with hUC-MSCs. A total of three time points of each group for sample collection after receiving hUC-MSCs injection: 1 day, 3 days and 7 days (*n* = 5 per group at every time point). To prevent confounding bias, the placement of each mouse cage was randomized.

### Flow cytometry assay of cells in mouse tissues

For single cell suspensions of the MLNs and spleen, organs were chopped and ground through a 70 μm nylon mesh and washed with an isotonic PBS buffer. After lysis of erythrocytes with erythrocyte lysate (Beyotime), the cells were washed several times with PBS buffer and resuspended in FACS containing 2% FBS.

First, before antibody incubation, unspecific staining was blocked with anti-mouse CD16/32 antibody (Biolegend, USA). And labelled with PBS 1:1000 dilution of dead-acting dye (Biolegend, Cat# 423,101) at 4 °C for 15 min. Second, stained with CD45-APC/fire 750 (Biolegend, Cat# 103,154), CD4 -FITC (Biolegend, Cat# 100,406) and CD25-PE (Biolegend, Cat# 113,704) in a dark room at 4 °C for 30 min. Third, punched and fixed with Transcription Factor Buffer Set (BD Pharmingen) at 4 °C for 20 min. Fourth, stained with RORγ-PE-610 (Thermo, Cat# 61-6981-82), and FOXP3-AF647 (Biolegend, Cat# 126,407) in a dark room at 4 °C for 30 min. Finally, suspension in FACS with 2% FBS. The stained cells were examined on a CytoFLEX S multicolor flow cytometer and analyzed with FlowJo software (BD Biosciences).

For flow cytometry detection of T cells in MLNs and spleen of mice. To examine the effect of hUC-MSCs treatment on T cells in MLNs and spleen, 20 mice were divided into four groups (*n* = 5): control mice, control mice injected with hUC-MSCs, DSS-induced colitis mice and DSS-induced colitis mice injected with hUC-MSCs. To examine the role of *TGFB1* knockdown on hUC-MSCs treatment in regulating T cells in MLNs, another 20 mice were divided into four groups (*n* = 5): control mice, DSS-induced colitis mice, DSS-induced colitis mice injected with hUC-MSCs^*NC*^ and DSS-induced colitis mice injected with hUC-MSCs^*TGFB1 KD*^. To prevent confounding bias, the placement of each mouse cage was randomized.

### Flow cytometry assay of cells cultured in vitro

For PBMC and mouse Th0 cells, which co-cultured with hUC-MSCs, the cell suspension was collected, respectively. Then, the supernatant was aspirated after centrifugation at 500 g for 5 min. Labelled with PBS 1:1000 dilution of dead-acting dye (Biolegend, Cat# 423,101) at 4 °C for 15 min. Subsequently, the dye was washed away with PBS and the supernatant was discarded after centrifugation at 500 g for 5 min.

For PBMC sample, stained samples with CD4-APC/fire 750 (Biolegend, Cat# 300,514), CD25-FITC (Biolegend, Cat# 53-0259-41), CD127-PE (Biolegend, Cat# 351,304) in a dark room at 4 °C for 30 min. Then, the antibody was washed away with PBS and the supernatant was discarded after centrifugation at 500 g for 5 min. Finally, suspension in FACS with 2% FBS.

For mouse Th0 cells, stained samples with CD4 -FITC (Biolegend, Cat# 100,406) and CD25-PE (Biolegend, Cat# 113,704) in a dark room at 4 °C for 30 min. Second, punched and fixed with Transcription Factor Buffer Set (BD Pharmingen) at 4 °C for 20 min. Third, stained with FOXP3-Cy5 (Thermo, Cat# 15-5773-80) in a dark room at 4 °C for 30 min. Fourth, the antibody was washed away with PBS and the supernatant was discarded after centrifugation at 500 g for 5 min. Finally, suspension in FACS with 2% FBS.

The stained cells were examined on a CytoFLEX S multicolor flow cytometer and analyzed with FlowJo software (BD Biosciences).

### Cell infection

hUC-MSCs were infected in vitro with Lentivirus for stable expression of RFP fluorescent protein (RFP-hUC-MSCs). Lentivirus were synthesized by Jiman Biotechnology (Shanghai, China). Infection according to the manufacturer’s protocol. Infection efficiency was examined by inverted fluorescence microscopy.

*TGFB1* small interfering RNA (siRNA) were synthesized by Jiman Biotechnology (Shanghai, China). The oligodeoxynucleotide sequences used in this study are presented in Table 1. Lipofectamine RNAiMAX infection reagent (Invitrogen, California, USA) was used for infection according to the manufacturer’s protocol. Infection efficiency was measured by qRT-PCR analysis.

**Table 1:** The oligodeoxynucleotide sequences of siRNA

Gene Name	Forward Sequence (5’-3’)	Reverse Sequence (5’-3’)
TGFB1_Homo	AGCGACTCGCCAGAGTGGTTA	GCAGTGTGTTATCCCTGCTGTCA
NC_Homo	UUCUCCGAACGUGUCACGUTT	ACGUGACACGUUCGGAGAATT

Lentivirus of *TGFB1* were added to complete medium and gently mixed (MOI = 15), then Polybrene was added to a final concentration of 8 µg/mL and gently mixed. Each of the above components was added in sequence. After 48 h of incubation at 37 °C, the lentivirus-containing medium was aspirated and replaced with fresh medium, and the cells were collected for subsequent in vivo experiments after 24–36 h of further incubation. Scarlet-Puro Lentivirus and Puromycin were synthesized by Jiman Biotechnology (Shanghai, China). Infection according to the manufacturer’s protocol. Infection efficiency was measured by qRT-PCR.

### Statistical analysis

To test for statistical significance, the Student’s t-test was used to compare two different groups. Comparisons between more than two groups were analyzed using two-way analysis of variance (ANOVA) with Dunnett’s test, or one-way ANOVA with Tukey’s test. Data are described by mean ± SEM, unless otherwise specified. *P* < 0.05 was considered statistically significant.

## Results

### Intraperitoneal treatment with hUC-MSCs alleviates colitis in mice

**Fig. 1:**
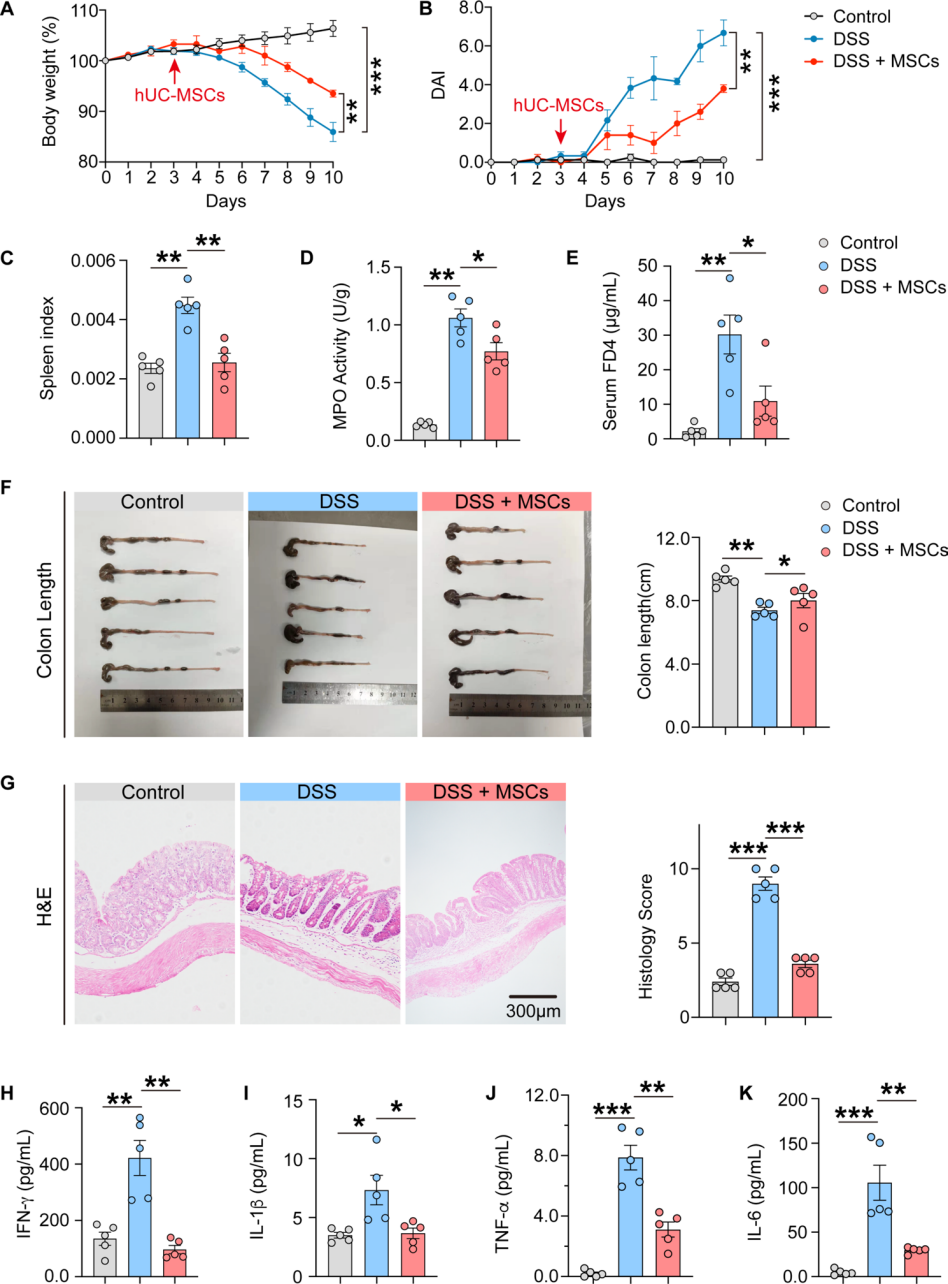
Intraperitoneal treatment with hUC-MSCs alleviated DSS-induced colitis in mice. (**A**) Weight loss was measured every day and expressed as the percentage change from day 0. (**B**) Disease activity index (DAI) score was monitored every day. (**C**) Spleen index of each group of mice on day 7 following the hUC-MSCs treatment. (**D**) The MPO activities in the colon of each group were determined on day 7 following the hUC-MSCs treatment. (**E**) Concentration of FD-4 in blood that penetrated through the intestinal epithelial barrier of each group were determined on day 7 following the hUC-MSCs treatment. (**F**) Macroscopic appearance (left) and length (right) of colon. (**G**) Colon histological sections stained with hematoxylin and eosin (H&E) of each group on day 7 following hUC-MSCs treatment. Scale bar: 300 μm. (**H-K**) The concentrations of IFN-γ (H), and IL-1β (I), TNF-α (J), IL-6 (K) in the serum of healthy control and DSS-induced colitis mice on day 7 following hUC-MSCs treatment were determined, respectively. Data are represented as the means ± SEM (*n* = 5). *P < 0.05, **P < 0.005, ***P < 0.001

Firs, the therapeutic effect of hUC-MSC intraperitoneal administration was compared to that of intravenous administration of hUC-MSCs in mice with DSS-induced colitis. As shown in Fig S1, intraperitoneal treatment with hUC-MSCs resulted in a significant amelioration in body weight (Fig. S1A), disease activity index (DAI) (Fig. S1B), spleen index (Fig. S1C), colon length and colon weight (Fig. S1, D-F) in colitis-afflicted mice, whereas intravenous administration did not yield improvement, as evidenced by the persistence of symptoms and phenotypes similar to those observed in the DSS group (Fig. S1, A-F). Therefore, intraperitoneal administration of hUC-MSCs was adopted in the following research. In addition to the notable improvements in body weight (Fig. 1A), disease activity index (DAI) (Fig. 1B), splenic index (Fig. 1C) and colon length (Fig. 1F), intraperitoneal administration of hUC-MSCs effectively mitigated the inflammatory injury to the intestinal barrier, as evidenced by reduced MPO activity (Fig. 1D) and decreased serum FD-4 permeability across the intestinal wall (Fig. 1E). Pathologically, intraperitoneal injection of hUC-MSCs was observed to ameliorate the morphology and architecture of the intestinal wall in DSS-induced mice (Fig. 1G). Furthermore, in colitis mice treated with hUC-MSCs, there was a notable decrease in serum levels of IFN-γ, IL-1β, TNF-α and IL-6 (Fig. 1, H-K), as well as reduced the mRNA expression levels of *Ifng*, *Il1b*, *Tnfa* and *Il6* in the colons (Fig. S1, G-J). These findings demonstrate that intraperitoneal injection of hUC-MSCs effectively alleviated colitis.

### The extensive distribution of hUC-MSCs in MLNs of colitis mice

**Fig. 2:**
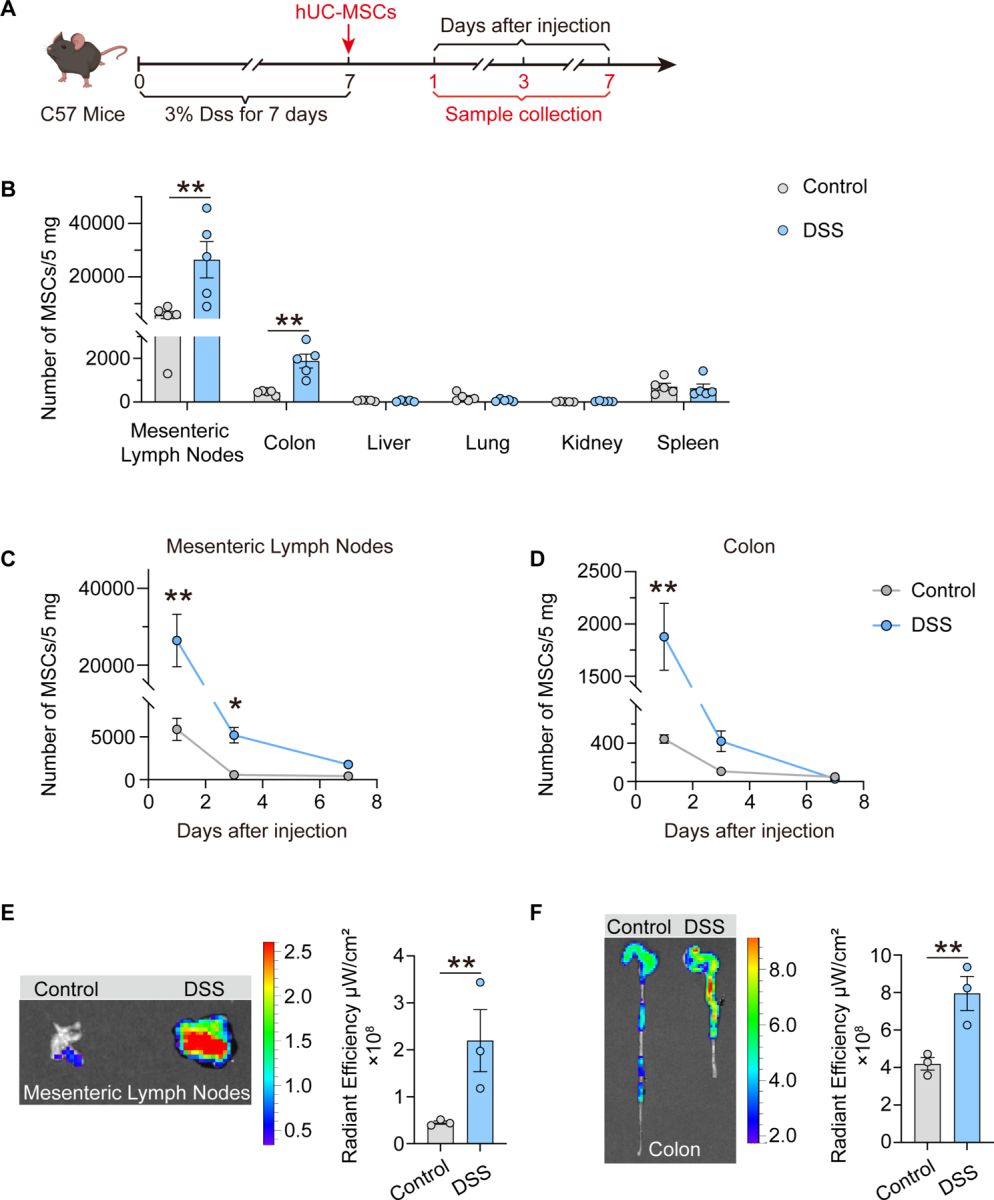
The extensive distribution of hUC-MSCs in MLNs of colitis mice. (**A**) Schematic timeline of DSS-induced colitis mice and intraperitoneal administration of hUC-MSCs. (**B**) hUC-MSCs numbers in the MLNs, colons, liver, lung, kidney and spleen of DSS-induced colitis and control mice at 1 day after hUC-MSCs intraperitoneal injection (*n* = 5). (**C and D**) hUC-MSCs numbers in the MLNs (C) and colons (D) of DSS-induced colitis and control mice at 1, 3 and 7 days after hUC-MSCs intraperitoneal injection (*n* = 5). (**E and F**) Fluorescence intensity of MLNs (E) and colons (F) at 1 day after intraperitoneal administration of RFP-hUC-MSCs (the hUC-MSCs expressing RFP fluorescent protein) is determined by IVIS Imaging System (*n* = 3). Data are represented as the means ± SEM. **P < 0.005, ***P < 0.001

An desensitize prerequisite for therapeutic effect of MSCs is their migration to the inflamed tissues or secondary lymphoid organs. To investigate this phenomenon, the biodistribution analysis of hUC-MSCs following intraperitoneal injection in DSS-induced colitis (Fig. 2A) and TNBS-induced colitis (Fig. S2A) mice was conducted, respectively. A significantly higher presence of hUC-MSCs was observed in the colon and MLNs of colitis mice compared to the healthy controls one day post-injection. And the concentration of hUC-MSCs in MLNs of colitis mice was much higher than that in colon and other tissues (Fig. 2B and S2B). The quantity of hUC-MSCs distributed within the MLNs and colons of both control mice and DSS-induced colitis mice peaked on day 1 and decreased over time. By day 3 post-intraperitoneal injection of hUC-MSCs, the quantity of hUC-MSCs in the MLNs of mice with DSS-induced colitis was significantly higher than that in control mice. At day 7, the distribution quantity had decreased to near the lower limit of detection (Fig. 2, C and D). Additionally, at 3 and 7 days post-injection of hUC-MSCs, the distribution of hUC-MSCs in various abdominal tissues of mice, including the stomach, spleen, liver, lung, and kidney, was found to be below the limit of detection. The findings suggest that hUC-MSCs exhibit rapid migration to the MLNs and the injury sites of colon within 1 day, followed by a significant elimination.

Visualization of RFP-hUC-MSCs (hUC-MSCs expressing RFP fluorescent protein) using IVIS demonstrated a notably higher fluorescence intensity in the colon and MLNs of DSS-induced colitis mice compared to the control mice (Fig. 2, E and F). There was no statistically significant variance observed in the fluorescence intensity levels within the kidneys, lungs, livers, and spleens between the two experimental groups (Fig. S2C). In order to further elucidate the specifics of the distribution pattern of hUC-MSCs following intraperitoneal injection in the colon, lymphatic vessels (LYVE1^+^) and epithelial cells (EPCAM^+^) were fluorescently labeled in the slices of colon tissues. The presence of hUC-MSCs in the colons of mice with colitis exceeded that in control mice, with a predominant localization within the crypt region and a notable accumulation within the lymphatic vessels (Fig. S2D). The above results indicate that hUC-MSCs not only homed to the colon but also distributed abundantly to MLNs.

### hUC-MSCs modulate Treg/Th17 cell equilibrium in MLNs through promoting local Treg differentiation

**Fig. 3:**
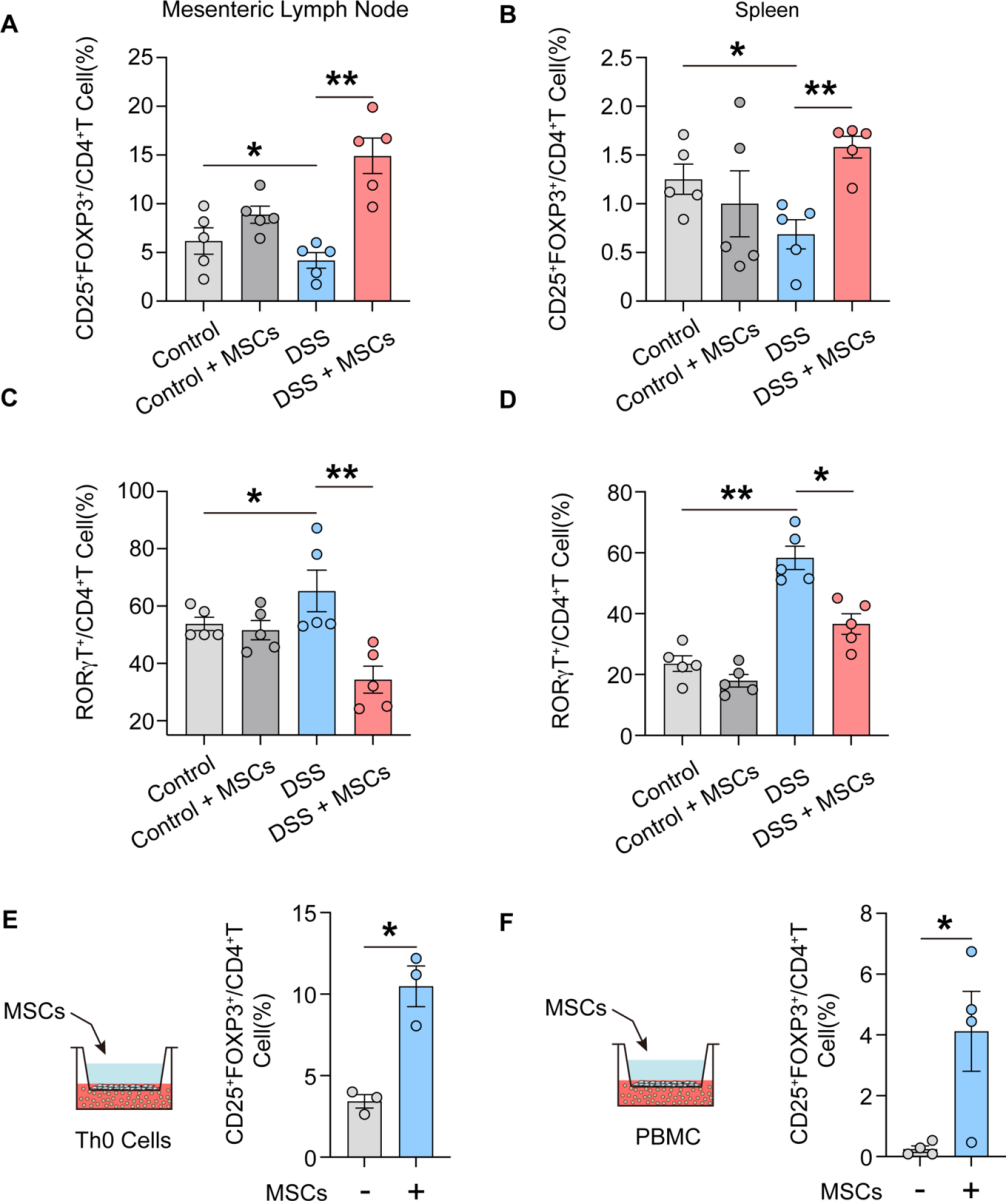
hUC-MSCs modulate Treg/Th17 cell equilibrium in MLNs and induce T cell differentiation into Treg cells. (**A** and **B**) Frequencies of Treg (CD25^+^ FOXP3^+^/CD4^+^) cells in MLNs (**A**) and spleen (**B**) of DSS-induced colitis and control mice at 3 days after hUC-MSCs intraperitoneal administration were detected by flow cytometry (*n* = 5). (**C** and **D**) Frequencies of Th17 (RORγT^+^/CD4^+^ T) cells in MLNs (**C**) and spleen (**D**) of DSS-induced colitis and control mice at 3 days after hUC-MSCs intraperitoneal administration were detected by flow cytometry (*n* = 5). (**E**) Frequencies of Treg cells of Th0 cells that co-culture with hUC-MSCs (*n* = 3). (**F**) Frequencies of Treg cells of PBMC that co-culture with hUC-MSCs (*n* = 4). Data are represented as the means ± SEM. **P < 0.005, ***P < 0.001

Colitis is characterized by dysregulation of Treg cells and Th17 cells in MLNs. To investigate the effect of hUC-MSCs treatment on Treg cells and Th17 cells in colitis MLNs, the frequency of Treg (CD25^+^ FOXP3^+^/CD4^+^) cells and Th17 (RORγt^+^/CD4^+^) cells in MLNs and spleen were evaluated at 3 days after intraperitoneal injection of hUC-MSCs. Compared to colitis model mice, hUC-MSCs treatment exhibited significantly higher levels of Treg cells in the MLNs and spleen (Fig. 3, A and B), while Th17 cells were notably reduced (Fig. 3, C and D). And the proportion of Treg cells in MLNs was much higher than that in spleen, indicating MLNs are critical sites for the up-regulatory effect of hUC-MSCs on Treg cells. To further investigate the potential mechanism by which hUC-MSCs may contribute to the increase in Treg cells in MLNs, we conducted co-culture experiments involving mouse spleen Th0 (naïve CD4^+^ T) cells and human PBMC with hUC-MSCs in Transwell, respectively. It was observed that co-culture with hUC-MSCs significantly promoted the differentiation of mouse spleen Th0 cells into Treg cells (Fig. 3E), as well as increased the proportion of Treg cells in human PBMC (Fig. 3F). The above results suggest that hUC-MSCs have the ability to modulate the Treg/Th17 cell balance in the MLNs of mice with colitis through the induction of Treg cell differentiation from naïve CD4^+^ T cells.

### hUC-MSCs upregulate the levels of TGF-β1 in MLNs of colitis mice

**Fig. 4:**
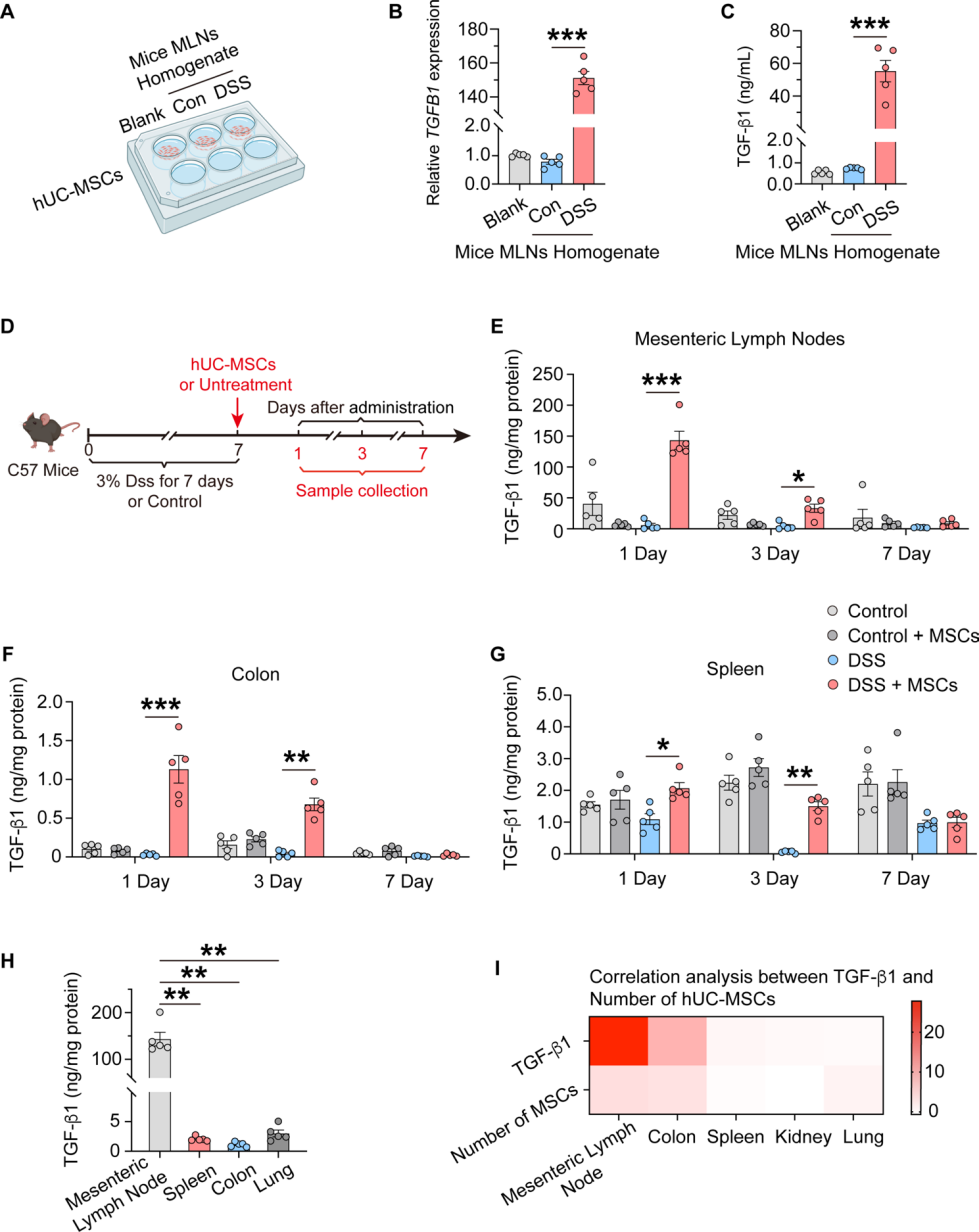
hUC-MSCs upregulate TGF-β1 levels in MLNs and colons. (**A**) Schematic representation of hUC-MSCs stimulated by MLNs homogenate from control mice or DSS-induced colitis mice for 24 h. (**B** and **C**) The *TGFB1* mRNA level (**B**) and the quantification of TGF-β1 secretion (**C**) from hUC-MSCs stimulated by MLNs homogenate from control mice or DSS-induced colitis (*n* = 5). (**D**) Schematic timeline of DSS-induced colitis mice and sample collection. (**E–G**) The concentrations of TGF-β1 in the MLNs (**E**), colon (**F**) and spleen (**G**) of DSS-induced colitis and control mice were quantified on days 1, 3, and 7 following either the intraperitoneal administration of hUC-MSCs or no treatment (*n* = 5). (**H**) The concentrations of TGF-β1 in different organs of DSS-induced colitis mice were quantified on day 1 following the intraperitoneal administration of hUC-MSCs (*n* = 5). (**I**) Correlation between TGF-β1 concentration and the numbers of hUC-MSCs distributed in MLNs, colons, spleen, kidney, lung of DSS-induced colitis mice were analyzed on day 1 following the intraperitoneal administration of hUC-MSCs. Data are represented as the means ± SEM. *P < 0.05, **P < 0.005, ***P < 0.001

It is known that cytokines are important for determining the fate of CD4^+^ T cells. For example, TGF-β1 and IL-2 are essential for Treg cell differentiation [29]. To further explore the impact of hUC-MSCs distribution in the MLNs on the local levels of TGF-β1. We first stimulated hUC-MSCs with MLNs homogenates from healthy control and DSS-induced colitis mice, respectively (Fig. 4A). The MLNs homogenates from colitis mice significantly enhanced the production of TGF-β1 by hUC-MSCs (Fig. 4, B and C). Furthermore, the mRNA level of *TGFB1* was up-regulated much higher than that of *TGFB2* and *TGFB3* in hUC-MSCs stimulated by MLNs homogenates from colitis mice (Fig. S4, A and B). Then, we examined the levels of TGF-β1 in MLNs, colon and spleen of healthy control and DSS-induced colitis mice at 1 day, 3 days and 7 days after intraperitoneal injection of hUC-MSCs (Fig. 4D). At 1 day and 3 days after intraperitoneal injection of hUC-MSCs, the levels of TGF-β1 levels in the MLNs (Fig. 4E), colon (Fig. 4F) and spleen (Fig. 4G) of colitis mice with hUC-MSCs treatment were all significantly increased compared to those in colitis mice. Moreover, the levels of TGF-β1 in MLNs were much higher than those in colon, spleen and lung at 1 day after intraperitoneal injection of hUC-MSCs (Fig. 4H). At 7 days after intraperitoneal injection of hUC-MSCs, TGF-β1 levels in serum of colitis mice with hUC-MSCs injection were significantly higher than in control mice and colitis model mice (Fig. S4C). There was no significant difference in the levels of TGF-β1 levels in kidneys (Fig. S4D) and lungs (Fig. S4E) between the control and colitis groups. Further analysis revealed that TGF-β1 concentration in MLNs was positively correlated with the numbers of hUC-MSCs distribution (Fig. 4I), suggesting hUC-MSCs may produce abundant TGF-β1 after they migrated into MLNs.

### *TGFB1* knockdown decreases the regulatory effect of hUC-MSCs on Treg cells in MLNs of colitis mice

**Fig. 5:**
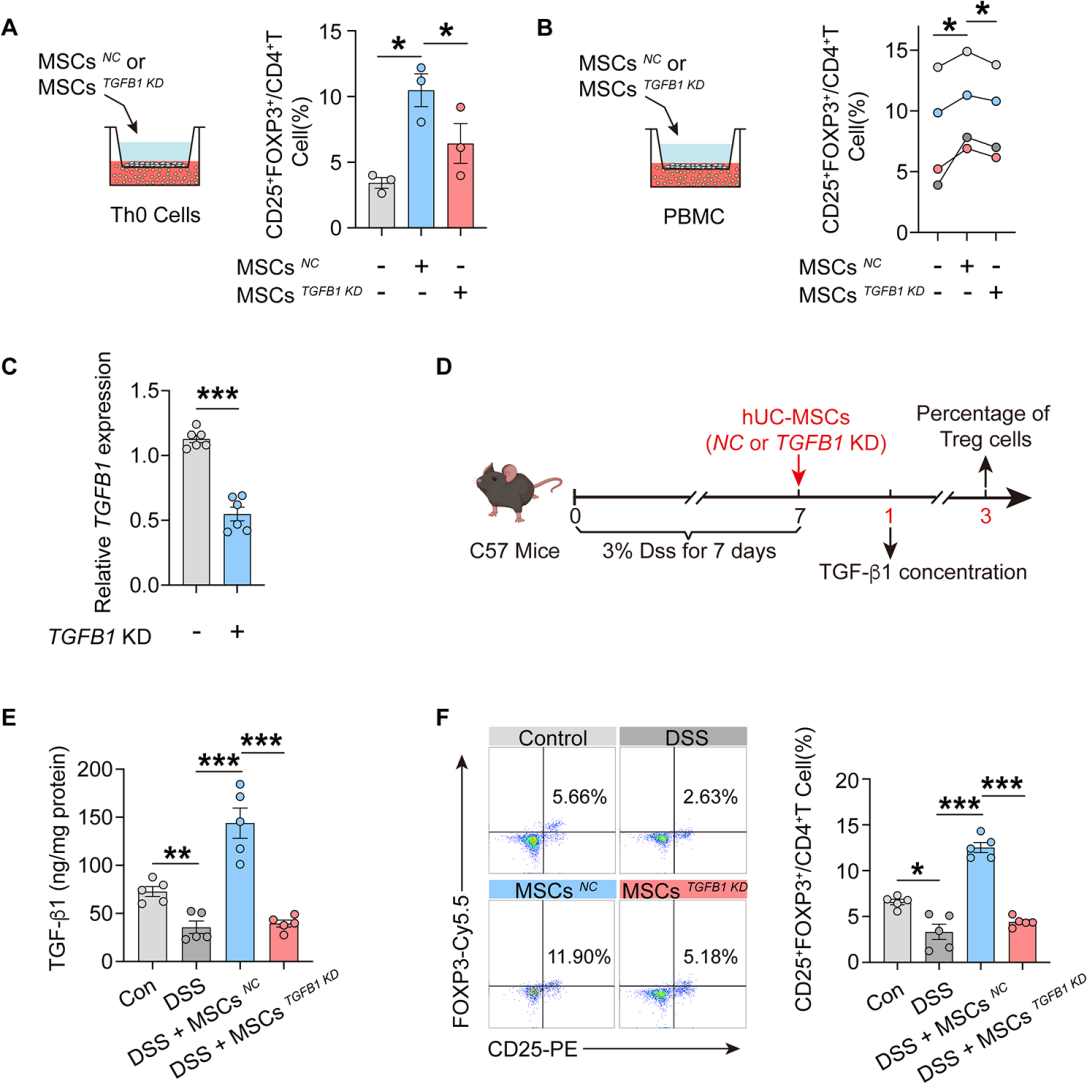
*TGFB1* knockdown decreases the regulatory effect of hUC-MSCs on Treg cells in MLNs of colitis mice. (**A**) Frequencies of Treg cells of Th0 cells that co-culture with hUC-MSC^*NC*^ or hUC-MSC^*TGFB1 KD*^ (*n* = 3). (**B**) Frequencies of Treg cells in human PBMCs that co-culture with hUC-MSC^*NC*^ or hUC-MSC^*TGFB1 KD*^ (*n* = 4). (**C**) The *TGFB1* mRNA expression in hUC-MSC^*NC*^ and hUC-MSC^*TGFB1 KD*^ were detected by qRT-PCR (*n* = 6). (**D**) Schematic timeline of DSS-induced colitis and intraperitoneal administration of hUC-MSCs (*NC* or *TGFB1* KD) in mice. (**E**) The concentrations of TGF-β1 in the MLNs of control mice, colitis mice and MSC-treated colitis mice at day 1 (*n* = 5). (**F**) Frequencies of Treg (CD25^+^ FOXP3^+^/CD4^+^) cells in MLNs from control mice, colitis mice and MSC-treated colitis mice at day 3 (*n* = 5). Data are represented as the means ± SEM. *P < 0.05, **P < 0.005, ***P < 0.001

Subsequently, hUC-MSC^*NC*^ (*Negative Control*) or hUC-MSC^*TGFB1 KD*^ (hUC-MSCs with *TGFB1* knockdown) were co-cultured with Th0 cells, resulting in a significant decrease in the differentiation of Th0 cells to Treg cells when co-cultured with hUC-MSC^*TGFB1 KD*^ compared to hUC-MSC^*NC*^ (Fig. 5A). Additionally, co-culturing hUC-MSC^*NC*^ or hUC-MSC^*TGFB1 KD*^ with human PBMCs showed a significantly lower proportion of Treg cells in PBMC co-cultured with hUC-MSC^*TGFB1 KD*^ compared to hUC-MSC^*NC*^ (Fig. 5B). To further investigate whether the increase in Treg percentage in MLNs of colitis mice was accomplished by TGF-β1 secretion of hUC-MSCs, the effect of hUC-MSC^*TGFB1 KD*^ treatment on Treg cells in MLNs of DSS-induced colitis mice were studied (Fig. 5, C and D). One day post-treatment with hUC-MSC^*NC*^, there was a significant increase in the concentration of TGF-β1 in the MLNs of colitis mice. Conversely, treatment with hUC-MSC^*TGFB1 KD*^ did not result in an enhanced concentration of TGF-β1 in the MLNs (Fig. 5E). This lack of TGF-β1 enhancement in the MLNs following hUC-MSC^*TGFB1 KD*^ treatment was associated with a failure to increase the percentage of Treg cells in the MLNs of colitis mice. In contrast, the hUC-MSC^*NC*^ group showed an increase in Treg cell numbers in the MLNs at day 3 after MSC treatment, indicating a potential role of hUC-MSC-derived TGF-β1 in T cell differentiation in MLNs (Fig. 5F). Meanwhile, there was no significant difference in the percentage of Treg cells in the spleens of colitis mice between the hUC-MSC^*NC*^ and hUC-MSC^*TGFB1 KD*^ groups (Fig. S5A).

### *TGFB1* knockdown reduces the therapeutic effects of hUC-MSCs on colitis

**Fig. 6:**
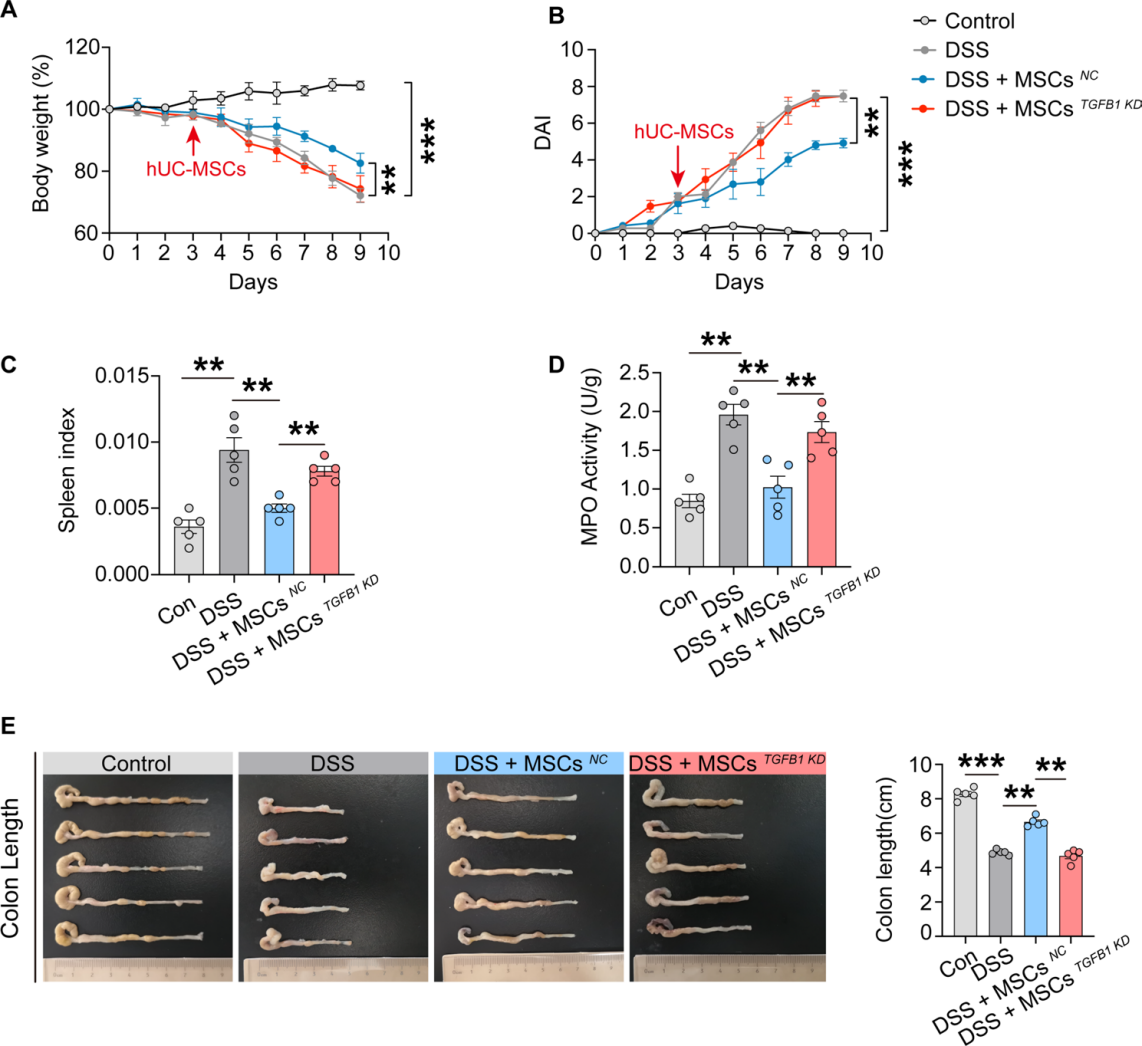
*TGFB1* knockdown reduces the therapeutic efficacy of hUC-MSCs on colitis. (**A**) Weight loss was measured every day and expressed as the percentage change from day 0 (*n* = 5). (**B**) Disease activity index (DAI) score was monitored every day (*n* = 5). (**C**) Spleen index of control mice, colitis mice and MSC-treated colitis mice at day 7 (*n* = 5). (**D**) The MPO activities in the colon of each group were determined using an MPO activity assay (*n* = 5). (**E**) Macroscopic appearance (left) and the length (right) of the colon (*n* = 5). Data are represented as the means ± SEM. **P < 0.005, ***P < 0.001

In comparison to treatment with hUC-MSC^*NC*^, treatment with hUC-MSC^*TGFB1 KD*^ did not yield the improvements in body weight (Fig. 6A), DAI (Fig. 6B), splenic index (Fig. 6C) and colon MPO levels (Fig. 6D) in colitis mice. Additionally, the colon length of colitis mice treated with hUC-MSC^*NC*^ exceeded that of both model colitis mice and colitis mice treated with hUC-MSC^*TGFB1 KD*^ (Fig. 6E). These findings suggest that the therapeutic efficacy of hUC-MSCs in colitis mice is diminished by the knockdown of *TGFB1*.

## Discussion

There are numerous reports on the biological properties of stem cells and their therapeutic effects on colitis [30, 31]. Systemically administered MSCs are still a widely used treatment for colitis [32], but numerous studies have reported that intravenous delivery of MSCs yields unsatisfactory results [33, 34]. For example, in a phase I–II clinical study that enrolled 13 patients with severe CD, only one patient achieved clinical remission [35]. In contrast, intraperitoneally injected MSCs have better therapeutic effects of colitis [10].

Abundant studies have shown that hMSCs have the property of homing to inflammation sites [36, 37], and the key prerequisite for MSCs to have a therapeutic effect is to reach the site of inflammation. It has been reported that intravenous hMSCs cannot distribute to the colon, which may be an important factor limiting the clinical efficacy of MSCs [23]. However systemically administered hMSCs were observed to migrate towards the inflamed colon, in another study with colitis mice [24, 38]. Discrepancies regarding homing experiments may result from different details in each protocol, including the number of cells, administered doses, the site of isolation, and the properties of the media [39–41]. Interestingly, we found that hUC-MSCs injected intraperitoneally in mice with colitis were distributed not only in the colon, but also in the MLNs, and the concentration in the MLNs was even much higher than that in the colon (Fig. 2). Our findings may provide a new insight into the in vivo distribution of intraperitoneally injected hUC-MSCs in colitis mice, and the regulatory effect of MSCs on immune cells in MLNs may be closely related to their therapeutic efficacy in treating IBD.

Treg cells may negatively regulate the activation of each of the major Th cell subtypes as well as other immune and inflammatory cells via cell-cell contact and production of soluble factors [42]. An imbalance of Th17 and Treg cells caused by Treg cells damage has been shown to play a significant role in IBD. And the balances between Th17 and Treg cells have been a key prognostic indicator in IBD immunopathogenesis [43]. Numerous in vitro and in vivo studies have shown that MSCs increase Treg number and activity and have the ability to specifically protect or induce Treg cells [44, 45]. For example, it has been shown that co-culture of allogeneic MSCs with CD4^+^ T cells induces human FOXP3^+^ CD25^high^ Treg cells [46]. This is consistent with our results that in vitro co-culture of MSCs significantly increased the number of Treg cells in mouse splenic Th0 cells or PBMCs. While it has been reported that MSCs treatment increases the number of Treg cells in MLNs, which in turn alleviates inflammatory pathological changes in the colitis phase and suppresses inflammatory cytokines secretion in the colon and serum [47, 48]. Our study demonstrated that the number of Treg cells significantly increased and the number of Th17 cells decreased in the MLNs of colitis mice after intraperitoneal injection of hUC-MSCs. This suggests that MLNs may be a critical site for the regulatory effect of hUC-MSCs on Treg cells and the subsequent efficacy on colitis.

Numerous studies have shown that TGF-β secreted by MSCs is involved in the immunomodulatory function of MSCs [49, 50]. TGF-β plays a key role in the generation and maintenance of Treg cells [51, 52], especially under inflammatory conditions. And it promotes the expression of Foxp3, thereby inducing the production of Treg cells [21]. Our study found that TGF-β1 concentration in MLNs was elevated and significantly higher than that in other tissues after hUC-MSCs intraperitoneal injection treatment. An imperative prerequisite for MSCs to regulate Treg cells by secreting TGF-β1 is to reach the site of inflammation. In the above study we have found the presence of mesenteric lymph node distribution of intraperitoneally injected hUC-MSCs, and by further study we have found a significant positive correlation between the concentration of TGF-β1 in MLNs and the number of hUC-MSCs. And *TGFB1* knockdown significantly impaired the ability of hUC-MSCs to induce Treg cells and treat colitis.

**Fig. 7:**
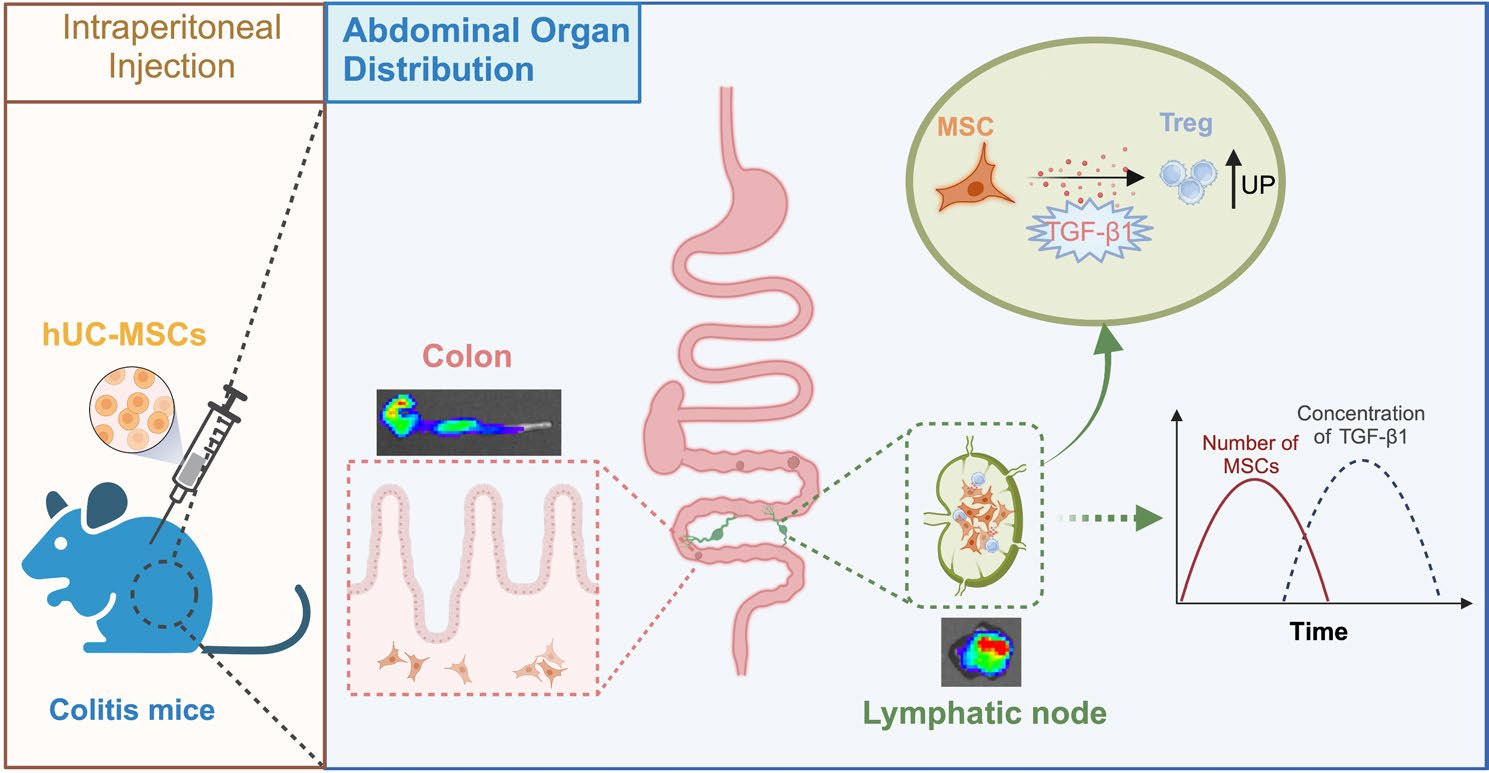
In colitis mice, hUC-MSCs migrate into MLNs quickly after intraperitoneal administration and subsequently upregulate Treg cells by secreting TGF-β1. hUC-MSCs after intraperitoneal administration not only homed to the colon but also distributed to the MLNs in significant quantities. This migration was accompanied by a notable increase in TGF-β1 concentration within the MLNs. Consequently, the upregulation of Treg cells in MLNs of colitis mice occurred as result of TGF-β1-mediated differentiation of T cells into Treg cells by hUC-MSCs, leading to a favorable therapeutic outcome for colitis

In conclusion, a close correlation was observed between the MLNs distribution properties of hUC-MSCs in MLNs and the therapeutic effect on colitis in mice, mediated by the induction of Treg cells through TGF-β1 produced by hUC-MSCs (See Fig. 7). MLNs may be a critical site for the regulatory effect of hUC-MSCs on Treg cells and the therapeutic effect on colitis.

## Electronic supplementary material

Below is the link to the electronic supplementary material.

Supplementary Material 1

## Data Availability

The authors confirm that all data underlying the findings are fully available.

## Abbreviations

hUC-MSCs: Human umbilical cord-derived mesenchymal stem cells
MSCs: Mesenchymal stem cells
Treg cell: T regulatory cell
Th17 cell: T helper 17 cell
PBMC: Peripheral blood mononuclear cell
RFP-hUC-MSCs: RFP fluorescently labelled hUC-MSCs
IBD: Inflammatory bowel disease
UC: Ulcerative colitis
CD: Crohn’s disease
GI: Gastrointestinal
GMSCs: Gingiva derived MSCs
PDLSCs: Periodontal ligament stem cells
MLNs: Mesenteric lymph nodes
TNBS: Trinitrobenzenesufonic Acid
DSS: Dextran sulfate
H&E: Hematoxylin and Eosin
DAI: Disease Activity Index
MPO: Myeloperoxidase
FD-4: Fluorescein isothiocyanate dextran-4
qRT-PCR: Quantitative real time polymerase chain reaction
siRNA: Small interfering RNA
Elisa: Enzyme-linked immunosorbent assay
IVIS: In vivo imaging system
PBS: Phosphate Buffered Saline
DMEM/F-12: Dulbecco’s Modified Eagle Medium/Nutrient Mixture F-12
TGF-β1: Transforming growth factor-β1
TNF-α: Tumor necrosis factor-α
IL-1β: Interleukin-1β
IL-6: Interleukin-6
IFN-γ: Interferon-γ
