# Correction: Mesenteric lymph nodes: a critical site for the up-regulatory effect of hUC-MSCs on Treg cells by producing TGF-β1 in colitis treatment

**DOI:** 10.1186/s13287-024-03867-1

**Published:** 2024-09-04

**Authors:** Qixiang Zhang, Zhu Zeng, Ning Wei, Yueyan Su, Jing Wang, Qi Ni, Yukai Wang, Jingwen Yang, Xiaoyan Liu, Huanke Xu, Guangji Wang, Yunlong Shan, Fang Zhou

**Affiliations:** 1 Key Laboratory of Drug Metabolism and Pharmacokinetics, Haihe Laboratory of Cell Ecosystem, State Key Laboratory of Natural Medicines, China Pharmaceutical University, Nanjing, China; 2 Jiangsu Renocell Biotech Co., Ltd, Nanjing, China

**Correction: Stem Cell Research & Therapy (2024) 15:190**

10.1186/s13287-024-03809-x

The original article mistakenly omitted a statement of co-Corresponding Authorship for both Guangji Wang and Yunlong Shan alongside Fang Zhou due to an oversight by the manuscript’s production team. The statement has since been restored.

