# Supplementary material for: Mesenteric lymph nodes: a critical site for the up-regulatory effect of hUC-MSCs on Treg cells by producing TGF-β1 in colitis treatment
Source: Stem Cell Res Ther. 2024 Jul 2;15:190. doi: 10.1186/s13287-024-03809-x (PMC11218300; doi:10.1186/s13287-024-03809-x)
Supplement: Supplementary file 1 — Supplementary Material 1 [file 13287_2024_3809_MOESM1_ESM.docx]

Supplementary information for

**Mesenteric Lymph Nodes: a critical site for the up-regulatory effect of hUC-MSCs on Treg cells by producing TGF-β1 in Colitis Treatment**

Qixiang Zhang^1^*, Zhu Zeng ^1^*, Ning Wei^1, 2^*, Yueyan Su^2^, Jing Wang^2^, Qi Ni^1^, Yukai Wang^1^, Jingwen Yang^1^, Xiaoyan Liu^1^, Huanke Xu^1^, Guangji Wang^1†^, Yunlong Shan^1†^, Fang Zhou^1†^

**Supplementary information lists:**

Materials and Methods

Fig. S1. Intraperitoneal treatment with hUC-MSCs alleviates colitis in mice.

Fig. S2. The extensive distribution of hUC-MSCs in MLNs of colitis mice.

Fig. S4. hUC-MSCs upregulate the levels of TGF-β1 in MLNs of colitis mice.

Fig. S5. *TGFB1* knockdown reduces the therapeutic effects of hUC-MSCs on colitis.

Table S1. Primers for quantitative real time polymerase chain reaction.

Reference

# Materials and Methods

## Induction of colitis by TNBS

Colitis was induced by TNBS (Sigma, USA) according to the method described previously^1^. All experiment procedures were conducted under general anesthesia (isoflurane, 3–5%, 0.5 L/min). BALB/c mice were anesthetized after a 24-h fast, then a 3.5-farthing (F) catheter fitted with a 1 mL syringe was inserted into the rectum, and TNBS (30 mg/mL) dissolved in 50% ethanol was introduced into the colon through the catheter for 100 µL. Control mice were injected with only the 50% ethanol solution.

## Distribution of hUC-MSCs

TNBS-induced colitis mice were randomized and injected intraperitoneally with hUC-MSCs (1 × 10^7^ cells/mice, 400 µL in volume). 30 mice were divided into two groups: control mice injected with hUC-MSCs and TNBS-induced colitis mice injected with hUC-MSCs. Three time points in each group for sample collection after receiving hUC-MSCs injection were: 1 day, 3 days, and 7 days (n=5 per group at every time point). At the indicated time points, animals of each group were euthanized. Different organs were collected for detecting the hUC-MSCs numbers. To prevent confounding bias, the placement of each mouse cage was randomized.

## mRNA Extraction and qRT‐PCR Amplification

Total mRNA was extracted with RNAiso Plus (Takara, Japan) based on the manufacturer’s protocol. Then, total mRNA was converted to complementary DNA (cDNA) by HiScript III RT SuperMix (Vazyme, China). The concentration and purity of total cDNA were detected by the Colibri Spectrophotometer (Berger, Germany). Then, the qRT-PCR (Quantitative real time polymerase chain reaction) assay of cDNA was performed in a volume of 15 µL that contained 7.5 µL qRT-PCR SYBR Green Mix (Bio-Rad, California, USA), 1 µL forward and reverse primers, and 200 ng template gDNA or cDNA diluted in water.

The Bio-Rad C1000 server system (Bio-Rad, California, USA) was used to determine mRNA levels. The 2^−ΔΔCt^ method was utilized for quantitative analysis. The mRNA levels were calculated with Beta‐actin (ACTB) or GAPDH as reference genes. Table S1 shows all mRNA primer sequences, which were synthesized by Sangon Biotech (Shanghai) Co., Ltd. (Shanghai, China).

## Fluorescence Imaging

Colon tissue sections are blocked at room temperature with 0.1% Triton X-100 in PBS and 5% donkey serum for 1 h. Tissue slides were incubated overnight at 4°C with diluted anti-mouse LYVE1 (1:400, Abcam) and anti-mouse EPCAM (1:400, Abcam), and finally stained with DAPI and mounted. The images were then observed using an Olympus Fluoview FV3000 confocal microscope (Olympus, Tokyo, Japan).

## Disease Activity Index (DAI) and Body Weight

Mice were monitored daily for body weight, presence/absence of gross blood in feces, and diarrhea. The disease activity index (DAI) was measured by assigning well-established and certified scores for parameters that are somewhat comparable to the clinical presentation of human IBD, as per Kang *et al*.^2^. DAI was the cumulative scores from the following three different parameters: weight loss score (0, none; 1, 1-5% body weight loss; 2, 5-10% body weight loss; 3, 11-15% body weight loss; 4, ˃ 15% body weight loss), diarrhea score (0, none; 2, mild; 4, severe watery), and presence of blood in feces (0, negative hemoccult; 2, occult bleeding; 4, gross bleeding). The total DAI score ranges from 0 (unaffected) to 12 (severe colitis).

## Measurement of Myeloperoxidase Activity

Myeloperoxidase (MPO) activity in the colon homogenate was determined using a Myeloperoxidase Assay Kit (Beyotime, China) according to the manufacturer’s instructions. Total protein concentrations were measured using a bicinchoninic acid (BCA) Protein Assay Kit (Thermo Fisher Scientific, USA) for calibration.

## Intestinal permeability test experiment

First, the mice were fasted overnight. Then, fluorescein isothiocyanate dextra-4 (FD-4) was dissolved in sterilised saline (50 mg/mL), protected from light, and the mice were weighed and garaged at 10 µL/g relative body weight. After 3 hours, 200 µL of blood was taken from the orbit and the serum was separated. Isolated serum was diluted in saline at 1:2 and 1:5 ratios. Dilute the remaining FD-4 for gavage with saline starting at a starting concentration of 0.1 mg/mL, 4-fold downward for 6 points. Finally, absorption at 520 nm under 490 nm excitation was performed simultaneously with the standard curve, plotted against the standard curve and reduced to the original concentration for averaging.

## Th0 cell acquisition and Co-culture with hUC-MSCs by Transwell

hUC-MSCs(1 × 10^5^ cells) were pre-inoculated on transwell inserts in 24-well plates. Naive CD4^+^ T cells were isolated from the spleens of mice. For naive T cell (Th0) culture, naive CD4^+^ T cells were cultured in a 48-well plate which was pre-coated with 2 µg/mL anti-CD3. For Treg cell culture, T cells were cultured with the addition of IL-2 (5 ng/mL) and TGF-β1 (20 ng/mL). After three days of culture and differentiation, hUC-MSCs implanted in transwell inserts were co-cultured with Th0 or Treg at a ratio of 1:10 for two days. After two days of co-culture, cells were collected for Flow Cytometry.

## PBMC Extraction and Co-culture with hUC-MSCs by Transwell

PBMC was extracted from fresh human blood samples by density gradient centrifugation, and was cultured in 1640 medium containing 10% calf serum (Gibco, Grand Island, USA). Then, hUC-MSCs implanted in transwell inserts were co-cultured with PBMC at a ratio of 1:10 for three days. After three days of co-culture, PBMC were collected for Flow Cytometry.

## Cell culture and reagents

hUC-MSCs, Human umbilical cord mesenchymal stem cells, were provide Jiangsu Renocell Biotech Co., Ltd. (Nanjing, China). hUC-MSCs were cultured with DMEM/F-12 mediums, which were supplemented with 10% fetal calf serum (Gibco, Grand Island, USA). Cells were cultured at 37°C in a humidified incubator containing 5% CO2.

# Supplementary Figures and Legends


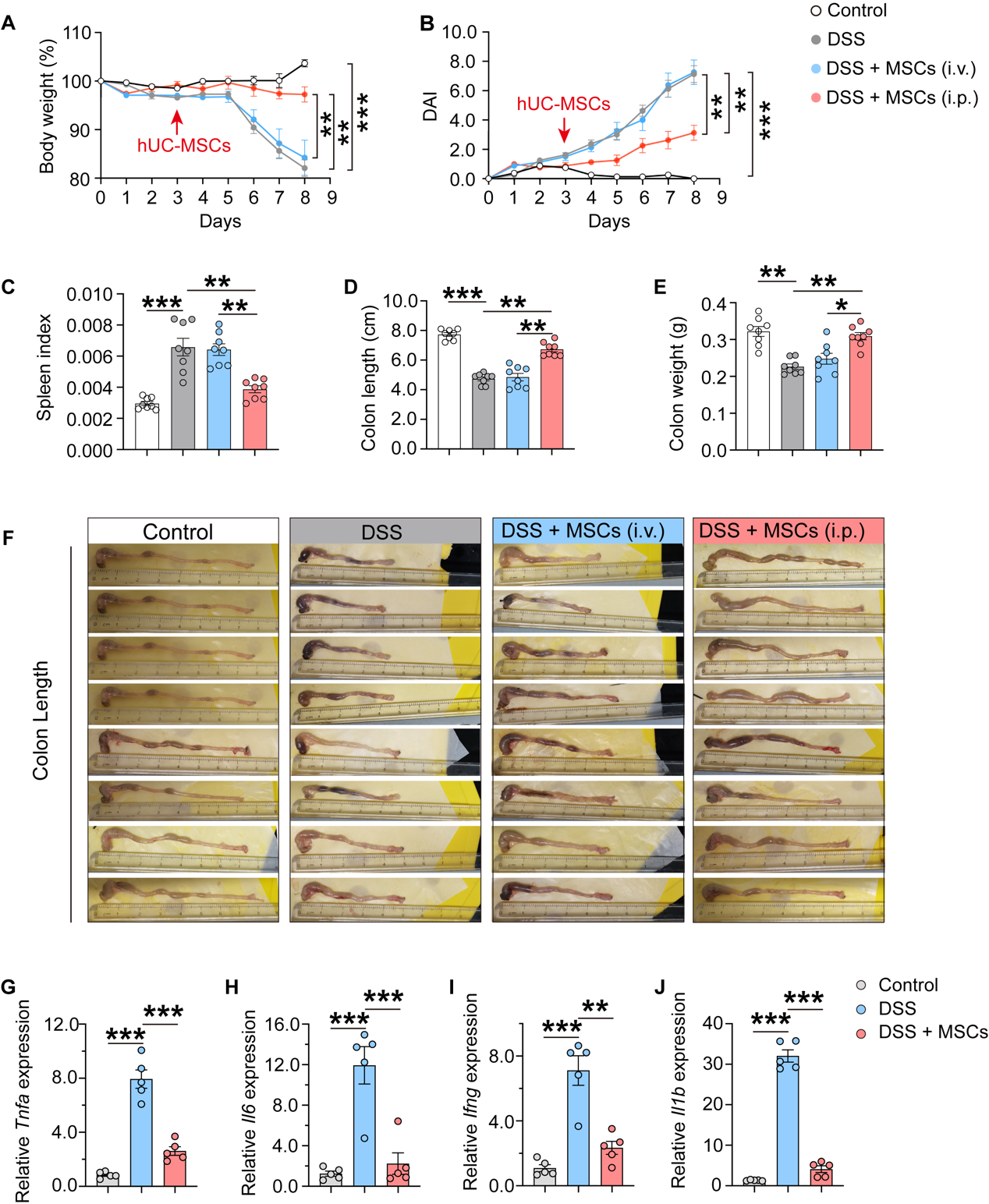


**Fig. S1. Intraperitoneal treatment with hUC-MSCs alleviates colitis in mice. (A)** Weight loss was measured every day and expressed as the percentage change from day 0 (n=8). **(B)** Disease activity index (DAI) score was monitored every day (n=8). **(C)** Spleen index of each group of mice on day 5 following hUC-MSCs treatment (n=8). **(D-F)** Colon length (D), colon weight (E) and macroscopic appearance (F) of each group mice on day 5 following hUC-MSCs treatment (n=8). **(G-J)** The mRNA expression level of *Tnfa* (G), *Il6* (H), *Ifng* (I), and *Il1b* (J) in the colon of healthy control and DSS-induced colitis mice on day 7 following hUC-MSCs treatment, respectively (n=5). Data are represented as the means ± SEM. **P* < 0.05, ***P* < 0.005, ****P* < 0.001.


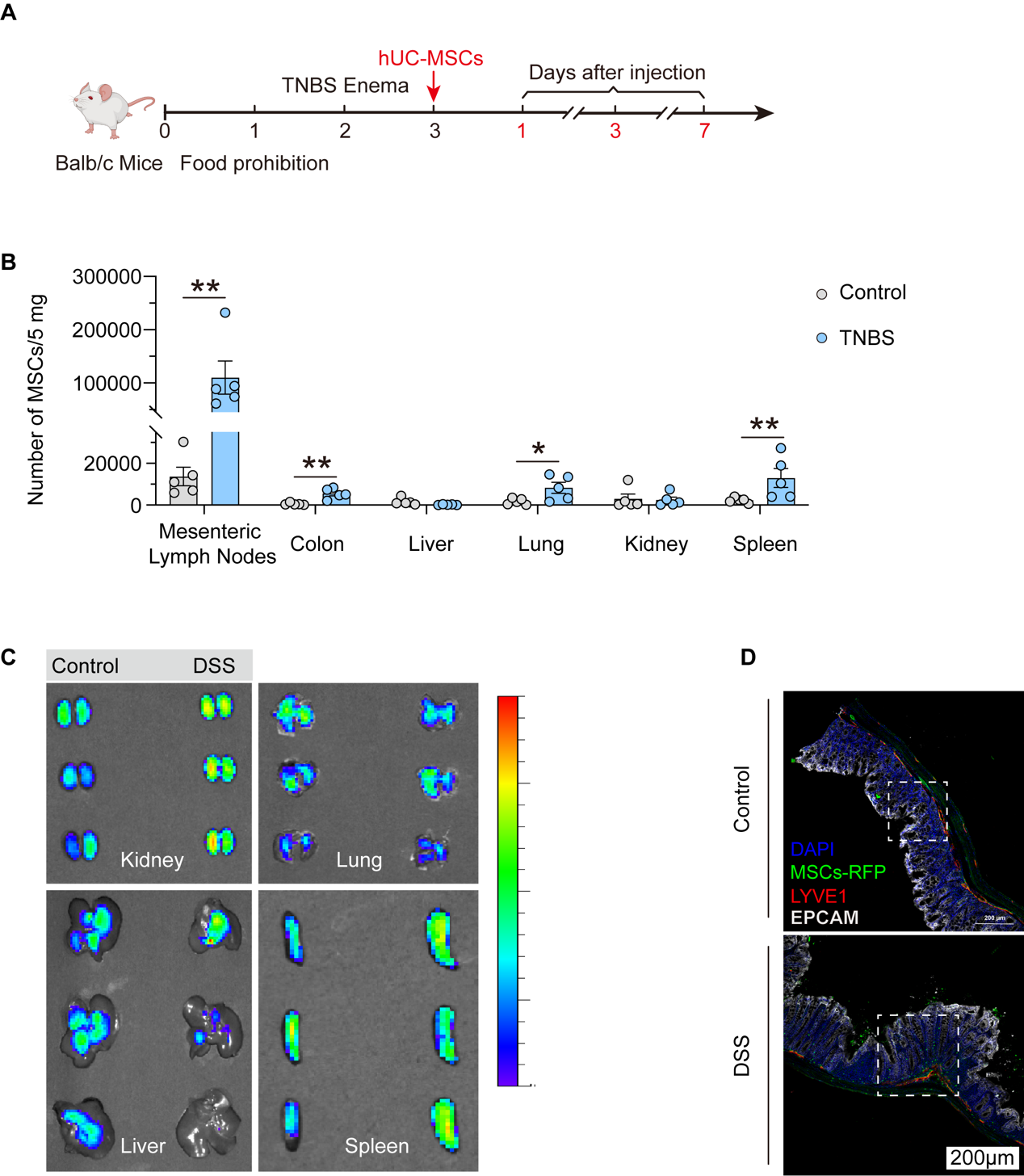


**Fig. S2. The extensive distribution of hUC-MSCs in MLNs of colitis mice. (A)** Schematic timeline of TNBS-induced colitis mice and intraperitoneal administration of hUC-MSCs. **(B)** hUC-MSCs numbers in the MLNs, colons, liver, lung, kidney and spleen of TNBS-induced colitis and control mice at 1 day after hUC-MSCs intraperitoneal injection (n=5). **(C)** Fluorescence intensity of kidney, lung, liver, spleen at 1 day after RFP-hUC-MSCs intraperitoneal injection is determined by IVIS Imaging System (n=3). **(D)** In fluorescent expression of lymphatic vessels (LYVE1), epithelial cells (EPCAM) and hUC-MSCs in the colon of DSS-induced colitis and control mice, hUC-MSCs in green, LYVE1 in red and EPCAM in silver (n=3). Scale bar: 200μm. Data are represented as the means ± SEM. **P* < 0.05, ***P* < 0.005, ****P* < 0.001.


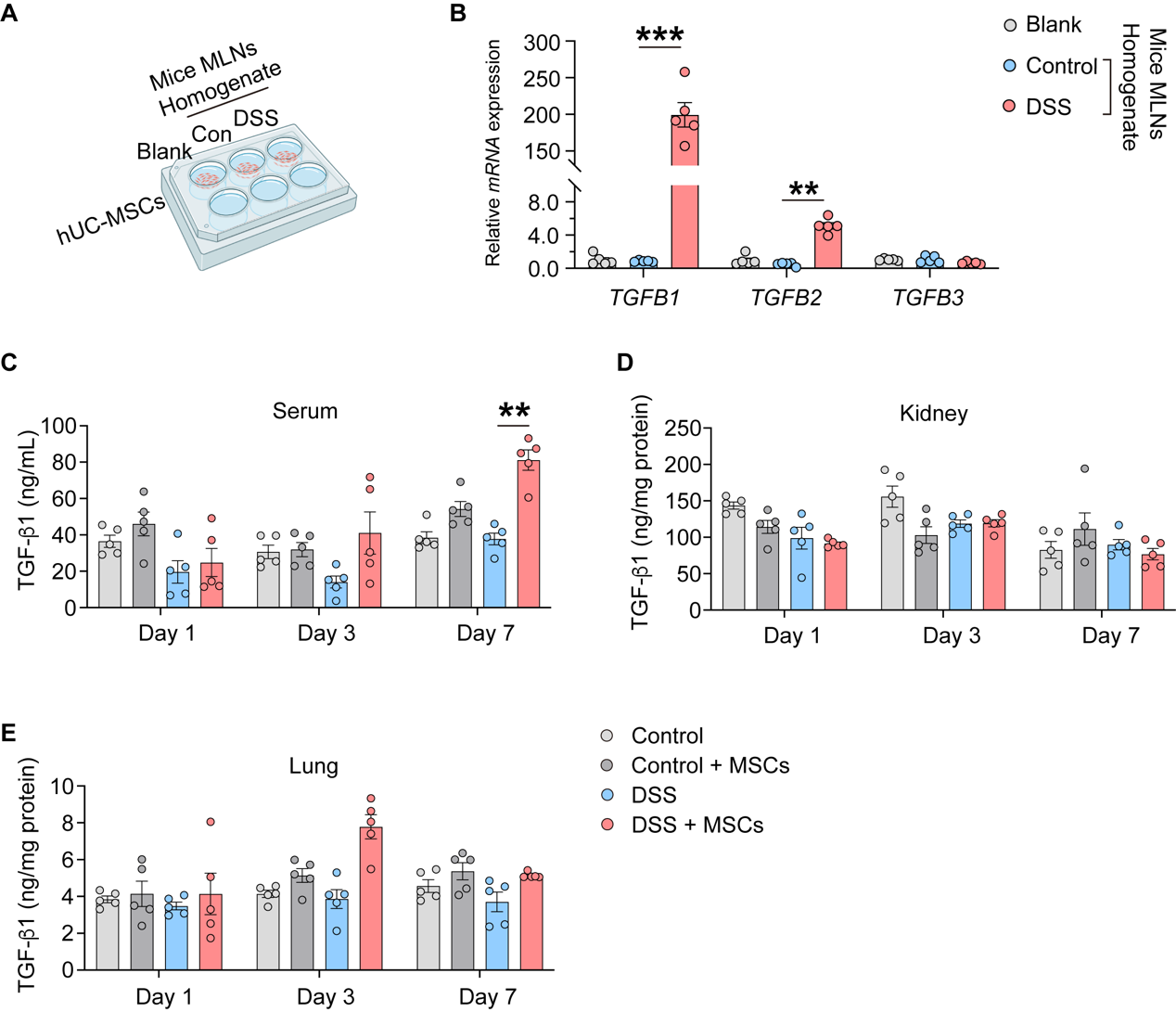


**Fig. S4. hUC-MSCs upregulate the levels of TGF-β1 in MLNs of colitis mice. (A)** Schematic representation of hUC-MSCs stimulated by MLNs homogenate from control mice or DSS-induced colitis mice for 24 hours. **(B)** The *TGFB1*, *TGFB2* and *TGFB3* mRNA level from hUC-MSCs stimulated by MLNs homogenate from control mice or DSS-induced colitis mice (n=5). **(C-E)** The concentrations of TGF-β1 in the serum (C), kidney (D) and lung (E) of DSS-induced colitis and control mice at 1 day, 3 days, and 7 days after hUC-MSCs intraperitoneal treatment (n=5). Data are represented as the means ± SEM. ***P* < 0.005.


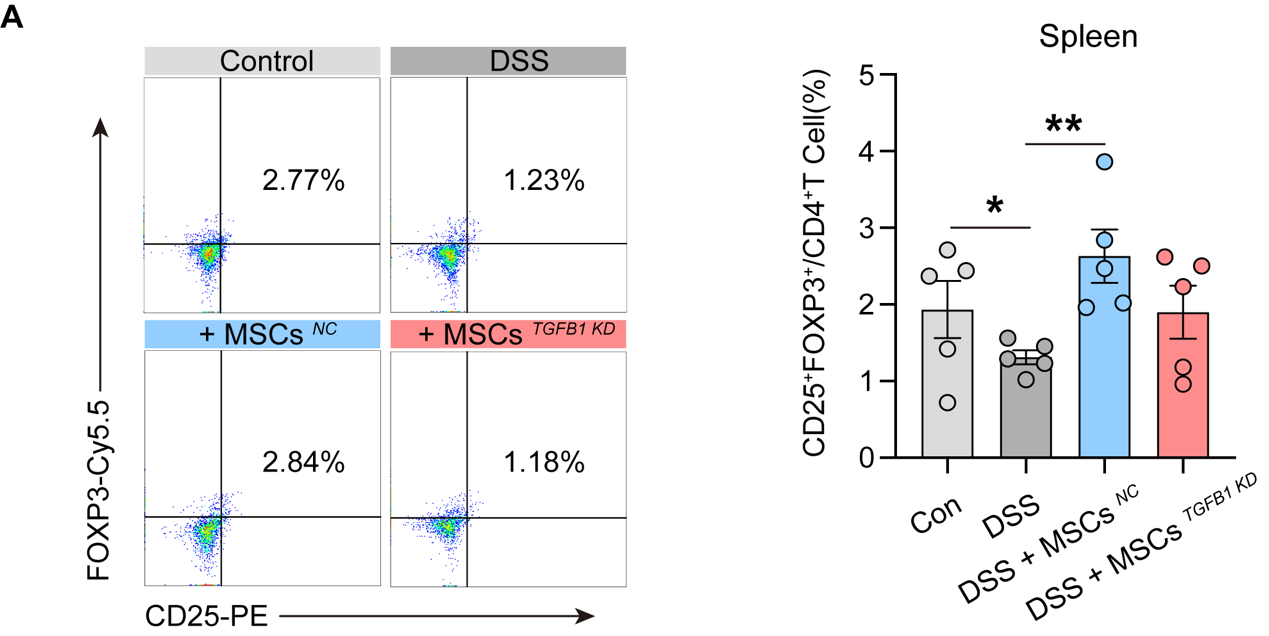


**Fig. S5. *TGFB1* knockdown reduces the therapeutic effects of hUC-MSCs on colitis. (A)** Frequencies of Treg (CD25^+^ FOXP3^+^/CD4^+^ T) cells in spleens from DSS-induced colitis and control mice at 1 day, 3 days, and 7 days after hUC-MSC *^NC^* or hUC-MSC *^TGFB1 KD^* intraperitoneal treatment were detected by flow cytometry (n=5). Data are represented as the means ± SEM. **P* < 0.05, ***P* < 0.005.

**Table S1.** Primers for quantitative real time polymerase chain reaction

| Gene Name | Forward (5’-3’) | Reverse (5’-3’) |
| --- | --- | --- |
| GAPDH_Homo | CATGTAGGCCATGAGGTCCACCAC | TGAAGGTCGGTGTCAACGGATTTGGC |
| TGFB1_Homo | GGCCAGATCCTGTCCAAGC | GTGGGTTTCCACCATTAGCAC |
| Actin_Mus | CTCCATCCTGGCCTCGCTGT | AACAGTCCGCCTAGAAGCAC |
| Tnfa_Mus | CCTCTCTCTAATCAGCCCTCTG | GAGGACCTGGGAGTAGATGAG |
| Il6_Mus | CTGCAAGAGACTTCCATCCAG | AGTGGTATAGACAGGTCTGTTGG |
| Ifng_Mus | GCCACGGCACAGTCATTGA | TGCTGATGGCCTGATTGTCTT |
| Il1b_Mus | GAAATGCCACCTTTTGACAGTG | TGGATGCTCTCATCAGGACAG |

## Reference

1 Ng, S. C. *et al.* Worldwide incidence and prevalence of inflammatory bowel disease in the 21st century: a systematic review of population-based studies. *Lancet* **390**, 2769-2778, doi:10.1016/S0140-6736(17)32448-0 (2017).

2 Huang, Y. F. *et al.* Anti-inflammatory effects of Brucea javanica oil emulsion by suppressing NF-kappaB activation on dextran sulfate sodium-induced ulcerative colitis in mice. *J Ethnopharmacol* **198**, 389-398, doi:10.1016/j.jep.2017.01.042 (2017).
